# Hydrophilicity controls thermodiffusion in alkylammonium chlorides

**DOI:** 10.1140/epje/s10189-026-00561-3

**Published:** 2026-02-24

**Authors:** Binny A. Rudani, Hartmut Kriegs, Simone Wiegand

**Affiliations:** 1 IBI-4, Research center Juelich, 52425 Juelich, Germany; 2 Institute for Light and Matter, University Cologne, 50939 Cologne, Germany

## Abstract

**Abstract:**

In this study, we examine the Soret effect of ammonium chloride ($\hbox {NH}_4$Cl) and its alkyl-substituted derivatives: dimethylammonium chloride (DMACl), ethylammonium chloride (EACl), and trimethylammonium chloride (TMACl) in aqueous solution using infrared thermal diffusion forced Rayleigh scattering. The Soret coefficient, $S_{\mathrm T}$, increases systematically with alkyl substitution, following the trend $\hbox {NH}_4$Cl $\ll$ DMACl < EACl $\ll$ TMACl, while hydrophilicity decreases correspondingly. Across the investigated temperature range ($15\!-\!45^\circ$C) and concentrations (1–4 mol/kg), $S_{\mathrm T}$ increases with both temperature and the degree of alkyl substitution. However, the concentration dependence varies among the salts. DMACl, EACl, and TMACl exhibit decreasing $S_{\mathrm T}$ with increasing concentration and are predominantly thermophobic; TMACl remains thermophobic under all conditions. In contrast, $\hbox {NH}_4$Cl shows a non-monotonic concentration dependence above $35~^\circ$C and is largely thermophilic. We discuss the origin of this minimum at elevated temperatures in relation to other aqueous salt systems that exhibit non-monotonic behavior of $S_{\mathrm T}$ with respect to concentration. Overall, each additional alkyl substitution decreases the temperature sensitivity of the Soret coefficient, $\Delta S_{\mathrm T}(\Delta T)$, consistent with reduced solute hydrophilicity. Furthermore, we observe a clear correlation between the thermal diffusion coefficient and the thermal expansion coefficient in these aqueous electrolyte solutions. This is consistent with the trends reported for nonpolar organic mixtures and aqueous solutions of non-ionic solutes. These findings highlight thermodiffusion as a sensitive probe for understanding how hydrophilicity and ion-specific interactions govern molecular transport in aqueous environments.

**Graphical abstract:**

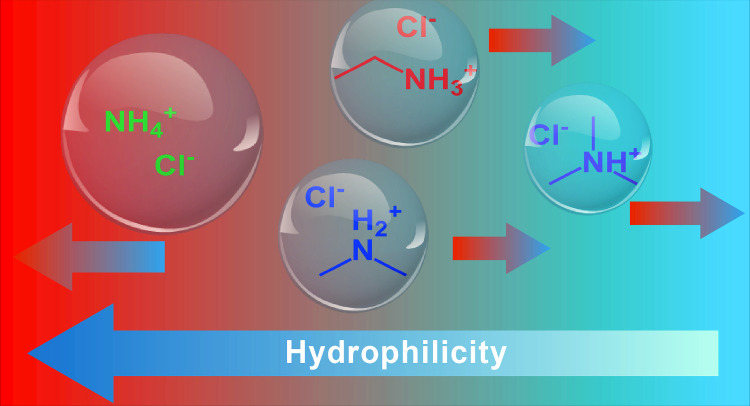

**Supplementary Information:**

The online version contains supplementary material available at 10.1140/epje/s10189-026-00561-3.

## Introduction

Thermodiffusion is the migration of solute molecules along a thermal gradient and is quantified by the Soret coefficient, $S_{\mathrm T}$. Thermodiffusion has numerous practical applications. It serves as a key mechanism in desalination [1], contributes significantly to petroleum reservoir management [2], and facilitates the formation of protein-based, cell-like proto-metabolic systems [3]. In energy storage and conversion, it influences the efficiency of thermogalvanic cells [4] and absorption chillers [5]. In biology and medicine, thermophoresis has become a powerful tool for probing biomolecular interactions, including protein–ligand binding and drug–target affinities [6, 7].

When it comes to aqueous systems, this process is sensitive to the nature of solute–water interactions, particularly to the structure and dynamics of the hydration layer surrounding the solute [8–11]. Owing to this sensitivity, thermodiffusion of aqueous systems has been widely investigated in recent years for various solutes such as colloids [12–14], polymers [15–17], biological molecules [3, 11, 18, 19] and small ionic and non-ionic solutes [5, 20–23].

Despite its broad utility, however, there is still no microscopic theory that can predict either the magnitude or the direction of the Soret coefficient. While mass, molecular shape, and moment of inertia primarily determine the thermodiffusive behavior of nonpolar substances, in polar and especially aqueous solutions, thermodiffusion is instead influenced by the solute’s hydrophilicity, as well as by pH and ionic strength [9].

In binary mixtures, solute transport arises from both concentration and temperature gradients and is explained by the flux expression

1 $$\begin{aligned} {\overrightarrow{J}} = - \rho D\overrightarrow{\nabla } c - \rho c\left( {1 - c} \right) {D_\mathrm{{T}}}\overrightarrow{\nabla } T\ \end{aligned}$$

where *c* is the solute mass fraction, *D* the Fickian diffusion coefficient, and $D_{\mathrm T}$ the thermal diffusion coefficient. At steady state, the balance of these two fluxes defines the Soret coefficient, $S_{\mathrm T} = D_{\mathrm T}/D$ [21, 22]. $S_{\mathrm T}$ can show positive or negative values, indicating the direction of solute migration under a temperature gradient. A positive $S_{\mathrm T}$ implies that solute molecules prefer the colder region, whereas a negative $S_{\mathrm T}$ means that solute accumulates in the warmer region.

The thermodiffusion response of ions in water is rather complex as it arises from intricate ion–solvent interactions that fundamentally govern the structural and dynamical properties of aqueous electrolytes. However, a microscopic understanding of these structural changes is still needed. The arrangement of water molecules around an ion is governed by the size, charge, and polarizability of the ion and gives rise to structured water layers whose organization is highly dynamic. These hydration shells reorganize continuously in response to concentration, temperature, pH, and counterions [24–26]. Hydrophobic hydration, particularly relevant for ions with nonpolar substituents, adds further complexity to ion–water interactions [27, 28]. Thus, in this work, we connect the well- characterized hydration of simple inorganic ions with the tunable chemistry of organic ions. A deeper understanding of ion–solvent interactions is essential for interpreting solvation thermodynamics and explaining ion-specific effects on diffusion, conductivity, and the optimization of electrolytes in cases such as the Seebeck effect for waste heat recovery, biomolecule transport, or prebiotic chemistry [28, 29].

The concentration and temperature dependence of the thermodiffusion behavior of aqueous salt solutions has been investigated through both experimental studies and simulations [10, 23, 30–40]. The concentration dependence of $S_{\mathrm T}$ for a salt in water is not uniform. It may increase, decrease or exhibit a minimum with increasing concentration. Similar to non-ionic molecules in water, the Soret coefficient generally increases with temperature. In some cases, the system exhibits thermophilic behavior at low temperatures and thermophobic behavior at high temperatures [22, 23], indicating a sign change in $S_{\mathrm T}$. As all salts are heavier than water, thermophobic behavior is expected if mass dominates, which is indeed the case for many salts [21, 32, 35, 37]. However, iodide salts exhibit negative Soret coefficients at low temperatures and concentrations [10, 40]. Additionally, few salts such as guanidinium thiocyanate, and many lithium salts, exhibit a negative $S_{\mathrm T}$ across a wide range of concentrations and temperatures [5, 10, 23, 34, 39, 41]. Hydration is believed to play a key role in determining the sign of the Soret coefficient and may be responsible for its negative values in certain systems. Studies suggest that strongly hydrated salts tend to accumulate on the warm side. The extent to which, besides hydrophilicity, a high charge density, as in lithium, is responsible for thermophilic behavior is still an open question. As temperature increases, the hydration shell weakens, leading to diminished thermophilic behavior. Because hydrogen bonding is highly temperature-dependent, this effect is reflected in the temperature sensitivity of thermodiffusive behavior [42, 43]. Recent studies have shown that the temperature dependence of the Soret coefficient $\Delta S_{\textrm{T}}$, defined as the difference in $S_{\mathrm T}$ between a high and a low temperature, correlates for diluted solutions directly with the hydrophilicity parameter $\log P$ [9, 22, 37]. The partition coefficient *P* denotes the concentration of a solute between an organic and an aqueous phase [44]. The calculation of $\log P$ is based on the assumption that the compound exists solely in its unionized form. In contrast, the distribution coefficient, $\log D_{\textrm{pH}}$, depends on pH, as it reflects the contributions of all solute species—ionized, partially ionized, and unionized—present at that specific pH. Note, that $\log D_{\textrm{pH}}$ is often simply written as $\log D$. Here, we use the subscript to avoid confusion with the collective diffusion coefficient *D*.

As mentioned at the beginning, the Soret coefficient of some salts shows a minimum as a function of concentration [10, 33, 34, 39, 45–47]. Since this phenomenon was reported [31, 45, 48–50], numerous attempts have been made to explain the observed behavior. Currently, two approaches are under discussion, both based on Lennard–Jones models, which are poorly suited to describing electrolyte solutions [10, 51]. The first approach links the minimum to the point where ion hydration shells begin to overlap, but relies on simplified ion-pair treatment and adjustable parameters [10]. Computer simulations of aqueous LiCl solutions also suggest that a strong hydration shell is crucial for understanding the minimum value of the Soret coefficient as a function of concentration [34]. The second study, conducted by Gittus and Bresme [51], relates the minimum of $S_\textrm{T}$ to a corresponding minimum in the thermodynamic factor. This relationship has been observed in simulations of Lennard–Jones particles, where kinetic effects are neglected. However, a recent analysis of various salt systems has shown that this purely thermodynamic approach fails to describe electrolyte solutions [40]. To date, no microscopic explanation has been provided for the minima of the Soret coefficient as a function of concentration.

**Table 1:** Properties of ammonium salts

Salt	$M_W$ / g/mol	$\log D_{\textrm{pH}}$	$N_{\textrm{don}}$	pH
$\hbox {NH}_4$Cl	53.49	-1.99	4	4.7
DMACl	81.54	-1.50	2	5.3
EACl	81.54	-1.40	3	5.4
TMACl	95.57	-1.35	1	5.5

$M_W$ is the molecular mass and $N_{\textrm{don}}$ is the number of hydrogen-bond donors. Details about the calculation of $\log D_{\textrm{pH}}$ can be found in the Supplementary Information Section S3. The pH of the salts was measured at 1 mol/kg

Among nitrogen-containing species, ammonium ($\hbox {NH}_4^+$) serves as a simple model system for probing ion–solvent interactions. With four N–H bonds, it forms a tetrahedral structure and a well-defined hydrogen-bonding environment, although the strength and rigidity of this shell remain debated [52]. A recent study by Ekimova [52] shows that $\hbox {NH}_4$ donates hydrogen bonds to four water molecules within its first solvation shell, while a fifth water molecule remains closely associated with the hydrated ion, influencing its rotational dynamics. However, their simulations suggest a weaker interaction that allows nearly free rotation of $\hbox {NH}_4^+$ in aqueous solution [52]. Extending beyond $\hbox {NH}_4^+$, alkylammonium halides represent an important class of salts that combine ionic and hydrophobic character within the same molecule, with hydrophobicity that can be tuned systematically [53]. Their hydration properties have been extensively studied using both experimental and simulation approaches [24, 54].

**Fig. 1:**
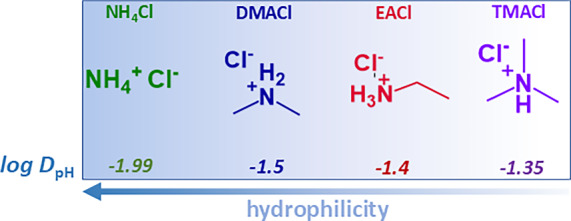
Chemical structures of the investigated ammonium salts along with their corresponding $\log D_{\textrm{pH}}$ values. A more negative $\log D_{\textrm{pH}}$ indicates greater hydrophilicity. The color of each structure corresponds to the representative color of the respective salt used throughout the figures in the manuscript

In this work, we investigate a well-defined series of ammonium salts, ammonium chloride ($\hbox {NH}_4$Cl), dimethylammonium chloride (DMACl), ethylammonium chloride (EACl), and trimethylammonium chloride (TMACl), to study their thermophoretic behavior using infrared thermal diffusion forced Rayleigh scattering (IR-TDFRS). The stepwise substitution of hydrogen atoms with alkyl groups introduces systematic variations in molecular size, mass, hydrophobicity, and charge distribution [24, 54]. The structures and key properties of these salts are presented in Fig. 1 and Table 1, respectively. An increase in alkyl substitution results in lower negative $\log D_{\textrm{pH}}$ values, which corresponds to a reduction in hydrophilicity. These progressive modifications provide a consistent framework for examining how alkyl substitution influences ion–water interactions and, consequently, thermophoretic behavior. We hypothesize that the degree of methyl substitution correlates directly with quantifiable changes in hydration, as reflected in the Soret coefficient.

## Experimental section

### Sample preparation

Ammonium chloride ($\ge$99% purity) was obtained from Thermo Fisher Scientific. Ethylammonium chloride, dimethylammonium chloride, and trimethylammonium chloride (each of $\ge$98% purity) were purchased from Sigma-Aldrich. All the salts were dissolved in distilled and deionized water (Millipore) to the desired concentrations. Note that all concentrations of the salts are below solubility limit. The resulting solutions were filtered through 0.2 $\mu$m filter and subsequently transferred into optical quartz Hellma cells with a 0.2 mm optical path length. Measurements were performed in duplicate using different cells, with freshly prepared samples for each individual system to ensure reproducibility.

### IR-TDFRS measurements

We employ IR-TDFRS, a laser-induced transient grating technique, to investigate thermodiffusion properties in aqueous systems [55]. The principle behind the method dates back to Thyagarajan and Lallemand [56] and was significantly further developed by Köhler [57]. In 2003, the method was validated against other thermal diffusion methods as part of a benchmark experiment [58]. In this technique, two intersecting infrared laser beams generate a spatially periodic holographic grating within the sample. Due to the intrinsic absorption of water at the selected wavelength, this optical pattern produces a corresponding temperature grating. The localized thermal modulation induces a periodic variation in the refractive index, forming a transient phase grating. A third probe beam is then diffracted by this grating, and the time-dependent intensity of the diffracted signal is measured. This intensity is directly proportional to the refractive index contrast and provides quantitative insights into the underlying thermal diffusion dynamics.

The heterodyne scattering intensity of the refracted read out beam is fitted with:

2 $$\begin{aligned} \zeta _{{\textrm{het}}} \left( t \right)  &   = 1 - \exp \left( { - \frac{t}{{\tau _{{\textrm{th}}} }}} \right) - A_0\left( {\tau - \tau _{\mathrm{{th}}} } \right) ^{ - 1} \nonumber \\  &   \quad \times \left\{ {\tau \left[ {1 - \exp \left( { - \frac{t}{\tau }} \right) } \right] - \tau _{{\textrm{th}}} \left[ {1 - \exp \left( { - \frac{t}{{\tau _{{\textrm{th}}} }}} \right) } \right] } \right\} . \nonumber \\ \end{aligned}$$

Here, $\tau _{th}=(D_{th}q^2)^{(-1)}$ and $\tau =(Dq^2)^{(-1)}$ are the lifetimes of the temperature and concentration grating, respectively, where *q*, $D_{th}$, and *D* in the equation denote the grating wave vector, the thermal diffusivity and the mutual diffusion coefficient, respectively. The steady-state amplitude $A_0$ in the above equation is given by

3 $$\begin{aligned} A_0 = \left( {\frac{{\partial n}}{{\partial c}}} \right) _{p,T} \left( {\frac{{\partial n}}{{\partial T}}} \right) _{p,c}^{ - 1} S_{\textrm{T}} c\left( {1 - c} \right) , \end{aligned}$$

The $S_\textrm{T}$ can be calculated from the amplitude *A*, if the contrast factors, namely the change in refractive index with temperature and concentration, $(\partial n/ \partial T)_{c,p}$ and $(\partial n/ \partial c)_{T,p}$, are known.

## Results and discussion

### Concentration dependence of $S_{\textrm{T}}$

**Fig. 2:**
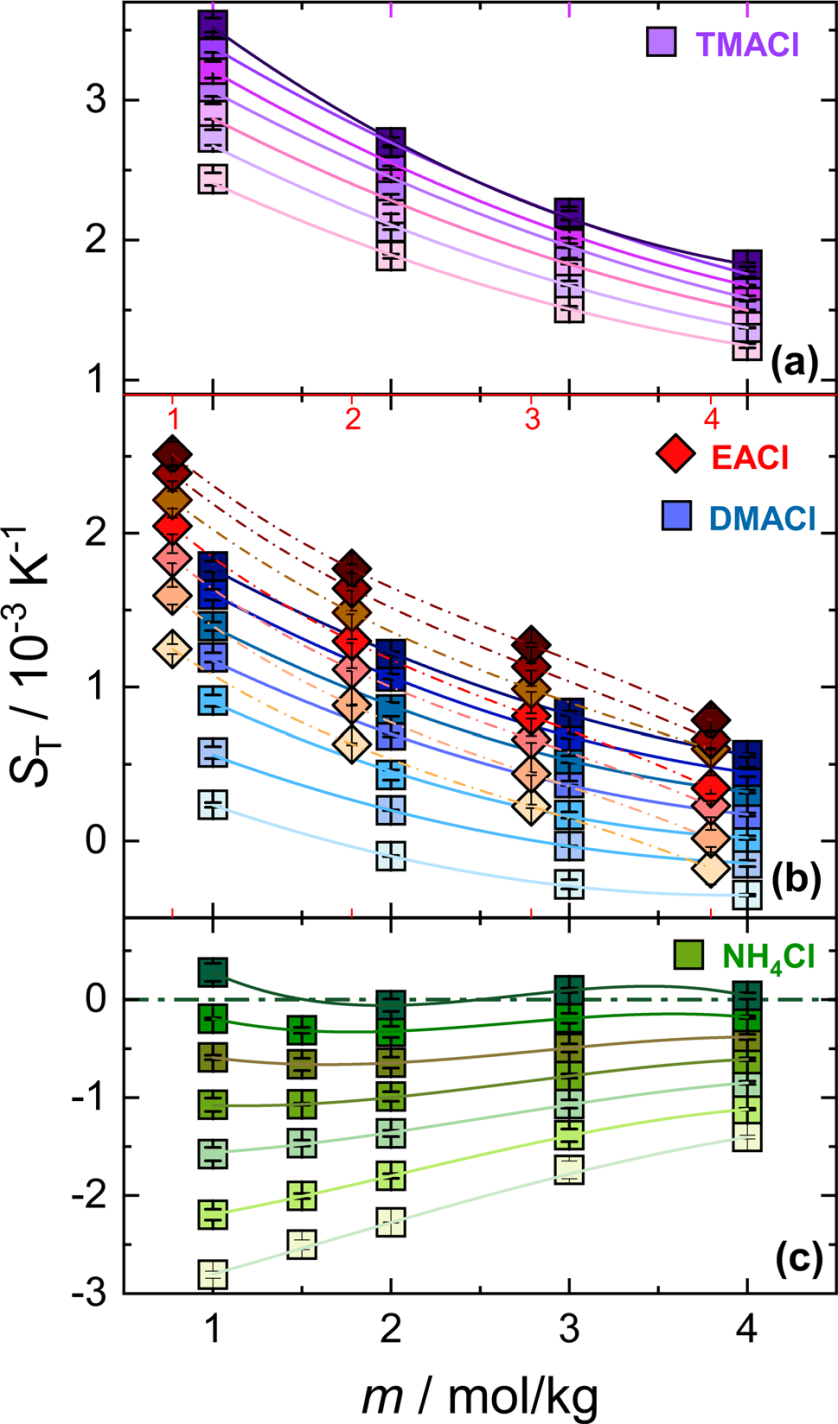
$S_{\mathrm T}$ of the investigated aqueous ammonium salt solutions as a function of concentration. Measurements were taken at temperatures ranging from $15^\circ$C (light colors) to $45^\circ$C (dark colors), with an increment of $5^\circ$C. For clarity, the top red x-axis corresponding to EACl is shown separately to facilitate direct comparison with DMACl. Error bars represent the standard deviation of the mean. Lines are included to guide the eye

The concentration dependence of $S_{\mathrm T}$ for all aqueous salt solutions is shown in Fig. 2. The lines are there to guide the eye. The Supplementary Information in section S1 describes the contrast factors required for the calculation of $S_{\mathrm T}$. Additionally, in Section S2 of the Supplementary Information the diffusion coefficient *D* and the thermal diffusion coefficient $D_{\mathrm T}$ are plotted for all temperatures and concentrations.

For the series of methyl- or ethyl-substituted ammonium chlorides: $S_{\mathrm T}$ decreases with increasing concentration, consistent with trends reported for other salt systems [30, 32, 40]. TMACl exhibits thermophobic behavior throughout the concentration range studied. While, EACl and DMACl are thermophobic over a wide range but show a sign change with concentration at low temperatures. To validate whether $S_{\mathrm T}$ exhibits a minimum with respect to concentration, we investigated DMACl at a concentration of 6 mol/kg, but the dependence on concentration remained monotonic (see Figure S5 in Section S4 in the Supplementary Information). Unlike the other salts, $\hbox {NH}_4$Cl exhibits primarily thermophilic behavior and a sign change at $45^\circ$C, the highest temperature investigated. Interestingly, $\hbox {NH}_4$Cl exhibits a distinct trend: at lower temperatures ($T < 30^\circ$C), $S_{\mathrm T}$ increases monotonically with concentration, whereas at $T > 30^\circ$C, the concentration dependence becomes non-monotonic, with a shallow minimum appearing near 1.5 mol/kg.

The magnitude of $S_{\mathrm T}$ at a given concentration increases with the degree of methyl substitution in ammonium chloride, following the trend $\hbox {NH}_4$Cl $\ll$ DMACl < EACl $\ll$ TMACl. For salts of same molecular weight, EACl exhibits a slightly larger magnitude of $S_{\mathrm T}$ compared to DMACl. Previous studies have shown that molecules with similar molecular weight but larger moments of inertia preferentially migrate toward the colder region [47, 59]. EACl, with its linear alkyl chain, possesses a larger moment of inertia than the branched DMACl, where two methyl groups are directly bound to the nitrogen center. Consequently, EACl displays a larger $S_{\mathrm T}$ magnitude.

Macaskill and Bates [54] reported osmotic and activity coefficients for monomethyl-, dimethyl-, and trimethylammonium chlorides in water. Below 2.3 mol/kg the activity coefficient decreases as the number of alkyl groups increases. Above the crossover concentration, however, the sequence is reversed. They attributed this behavior to dipole–ion interactions dominating at low concentrations and hydrophobic interactions at higher concentrations. Although thermodiffusion behavior is generally sensitive to changes in interactions, we observe a monotonic increase in the Soret coefficient as the number of alkyl groups increases. This indicates that mass dominates the behavior at this point.

**Fig. 3:**
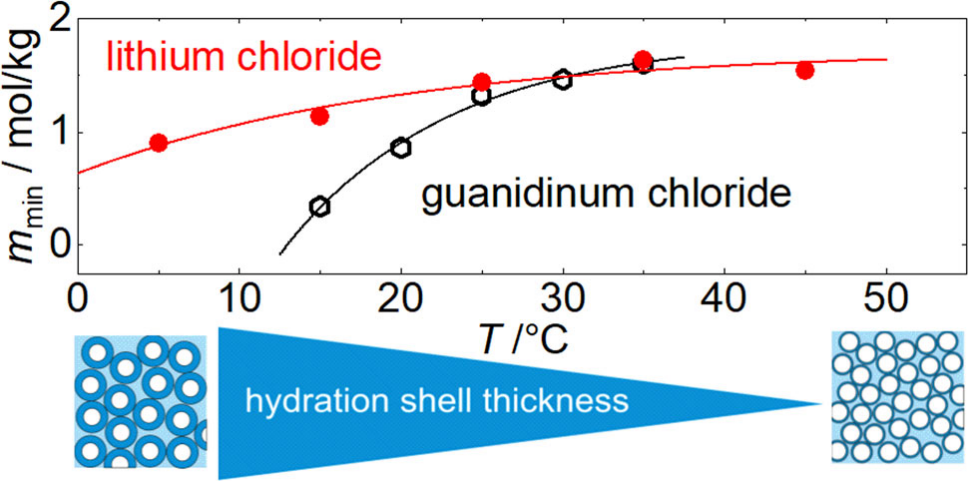
Concentration $m_\textrm{min}$ at which $S_{\textrm{T}}$ has a minimum with concentration increases with temperature in aqueous lithium chloride (LiCl) and guanidinium chloride (GdmCl) solutions [23, 39]. The lines are exponential fits of the data points. Assuming this minimum reflects random close packing of hydrated spheres [10], a more flexible or thinner hydration layer at higher temperatures shifts the minimum to higher concentrations, as illustrated

As shown in Fig. 2, a very shallow minimum in the Soret coefficient develops for $\hbox {NH}_4$Cl above $35^\circ$C, a temperature at which hydrogen bonds are already weakening. This observation is consistent with the minimum in the solutal expansion coefficient reported at approximately $35^\circ$C [60]. Above this temperature, the solutal expansion coefficient increases more rapidly because the voids previously created by the hydrogen-bond network can no longer be occupied by $\hbox {NH}_4$Cl; as a result, the density does not rise as strongly.

A similar minimum in the Soret coefficient is also observed for cesium iodide (CsI) above $30^\circ$C. Unfortunately, to the best of our knowledge, no experimental density data exist for aqueous CsI solutions, preventing us from verifying the corresponding solutal expansion coefficient for this system. Another possible explanation for why the minimum only becomes noticeable at higher temperatures is that it occurs at lower concentrations at lower temperatures, shifting towards higher concentrations as the temperature increases. This behavior has been observed for lithium chloride (LiCl) and guanidinium chloride (GdmCl), as illustrated in Fig. 3. A heuristic explanation is that at lower temperatures, the hydration layer is more extensive than at higher temperatures. Consequently, the condition for random close packing is reached only at higher temperatures, as schematically depicted in the cartoon in Fig. 3 [10].

### Temperature dependence of $S_{\textrm{T}}$

**Fig. 4:**
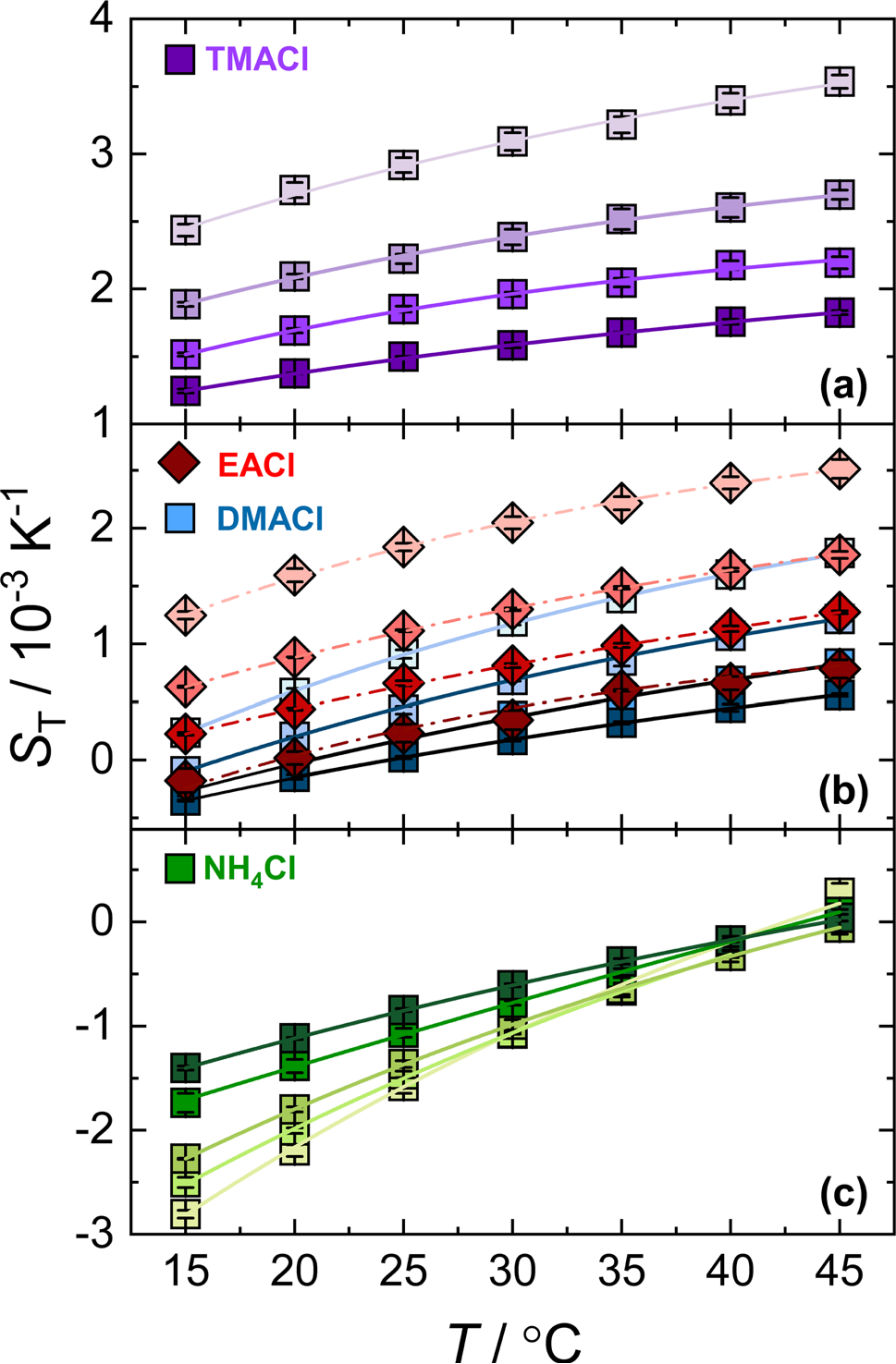
$S_{\textrm{T}}$ of aqueous ammonium salt solutions as a function of temperature at concentrations ranging from 1 mol/kg (light color) to 4 mol/kg (dark color). Error bars represent the standard deviation of the mean. Lines correspond to fits based on Eq. 4; fitting parameters are summarized in Table S1 in Section S5 of the Supplementary Information

Thermodiffusion has been observed to exhibit a strong dependence on temperature for both non-ionic and ionic solutes [9, 21, 37]. Next, we examined the temperature dependence of $S_{\textrm{T}}$ for all ammonium salts, which is shown in Fig. 4. For all systems, $S_{\mathrm T}$ increases with an increase in temperature. However, the $S_{\mathrm T}$ of $\hbox {NH}_4$Cl converges at high temperature. Such crossing points are typical of highly hydrophilic solutes, whether they are non-ionic or ionic, and have been observed in experiments and simulations [20, 39, 61]. In less hydrophilic systems, however, the change in $S_{\textrm{T}}$ with respect to temperature for different concentrations is less pronounced, meaning that no crossing point can be observed.

The lines are fitted according to equation 4, which describes the temperature dependence of an aqueous system. The fitted parameters are listed in Table S1 in Section S5 of the Supplementary Information. It is an empirical equation proposed by Iacopini and Piazza [12], but in a slightly modified form,

4 $$\begin{aligned} S_{\mathrm T}(T)\;=\;S_{\mathrm T}^\infty \;+\;A\;\textrm{exp}\;\left( \frac{-T}{T_0}\right) \end{aligned}$$

where $S_{\mathrm T}^\infty$ denotes the high-temperature limit of $S_{\mathrm T}$ and $T_0$ characterizes the curvature of $S_{\mathrm T}$. The amplitude *A* reflects the degree of temperature sensitivity. Unfortunately, in some cases, the temperature dependence of $S_{\mathrm T}$ is almost linear, and the obtained fitting parameters have significant uncertainties. A more reliable measure of temperature sensitivity is therefore given by $\Delta S_{\textrm{T}}(\Delta T)$, defined as the difference in $S_{\mathrm T}$ between a high and a low temperature [23]. For this investigation we used $T_{\textrm{high}}=45^\circ$C and $T_{\textrm{low}}=15^\circ$C. Note, that the same temperature range should be used if you want to compare different systems. Many recent studies have related $\Delta S_{\textrm{T}}(\Delta T)$ and the amplitude *A* in Eq. 4 to the number and the strength of hydrogen bonds [9, 11, 37].

**Fig. 5:**
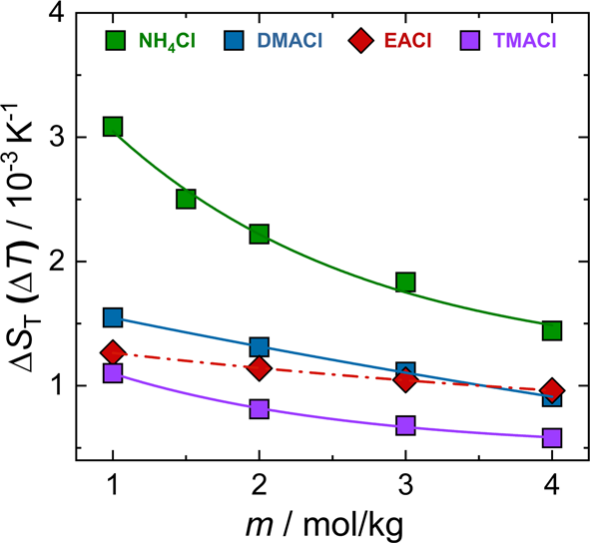
$\Delta S_{\textrm{T}}(\Delta T)$ plotted as a function of increasing ammonium salt concentration. The lines are guide to eye

The corresponding $\Delta S_{\textrm{T}}(\Delta T)$ versus concentration plot is shown in Fig. 5. For all salts, $\Delta S_{\textrm{T}}(\Delta T)$ decreases with increasing concentration, with the steepest decline observed for $\hbox {NH}_4$Cl, while the substituted ammonium salts exhibit a more moderate decrease. In the case of EACl, the slope of $\Delta S_{\textrm{T}}(\Delta T)$ is less steep than that of other salts, probably due to the bulky alkyl side group. However, at 4 mol/kg, its $\Delta S_{\textrm{T}}(\Delta T)$ matches the DMACl value.

The variation in $S_{\textrm{T}}$ of DMACl with temperature at different concentrations is shown in Figure S5 in Section S4 in the Supplementary Information. The inset shows the temperature sensitivity, defined as $S_{\textrm{T}}(\Delta T) = S_{\textrm{T}}(45^\circ \textrm{C}) - S_{\textrm{T}}(15^\circ \textrm{C})$, plotted against concentration. The results reveal that $S_{\textrm{T}}$ increases consistently with both concentration and temperature. While, the $\Delta S_{\textrm{T}}(\Delta T)$ decreases with an increase in concentration. Thus, we expect no noticeable deviation from the overall trend.

### Influence of the solute’s hydrophilicity on the thermodiffusion

Previous studies have shown a direct correlation between the temperature sensitivity of $S_{\textrm{T}}$, expressed as $\Delta S_{\textrm{T}}(\Delta T)$, and the hydrophilicity of solutes, typically represented by $\log P$ for non-ionic solutes [9]. In the present study we investigate aqueous salt solutions, which are better described by the distribution coefficient, $\log D_{\textrm{pH}}$. As mentioned earlier this coefficient accounts for both ionized and neutral species at a given pH, describing the hydrophilicity of the solute. It will be used to correlate with $\Delta S_{\textrm{T}}(\Delta T)$. In general, the most hydrophilic molecule has the most negative $\log P$ or $\log D_{\textrm{pH}}$ value. Additional information on the calculation of $\log D_{\textrm{pH}}$ is available in the Supplementary Information in Section S3.

**Fig. 6:**
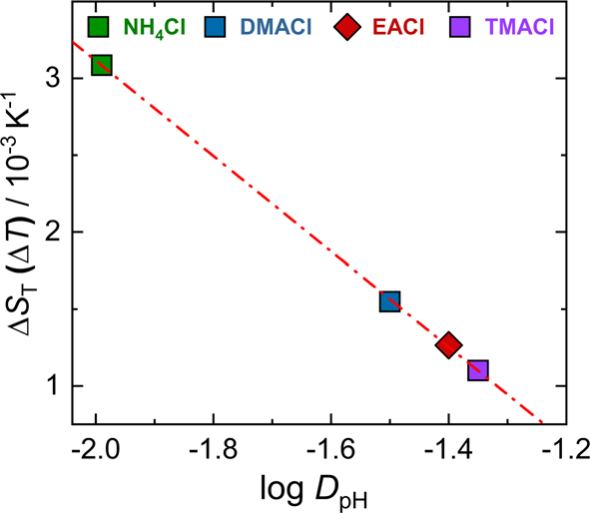
$\Delta S_{\textrm{T}}(\Delta T)$ for 1 mol/kg salt solutions plotted against $\log D_{\textrm{pH}}$

Figure 6 presents the temperature sensitivity of $S_{\textrm{T}}$, $\Delta S_{\textrm{T}}(\Delta T)$ as a function of $\log D_{\textrm{pH}}$. The data show a linear decrease in $\Delta S_{\textrm{T}}(\Delta T)$ with successive alkyl substitution on ammonium chloride salts, following the trend: $\hbox {NH}_4$Cl > DMACl > EACl > TMACl. As methyl or ethyl groups are successively substituted onto the ammonium ion, the number of hydrogen-bond donor sites $N_{\textrm{don}}$ decreases, while hydrophobic alkyl groups are introduced that perturb the local structure of water. This leads to changes in effective surface charge density of the solute molecule: $\hbox {NH}_4$Cl, which lacks bulky substituents, has the highest surface charge density and therefore the strongest ion–water electrostatic interactions. Introducing methyl or ethyl groups reduces the effective surface charge density through steric shielding and delocalization of positive charge over the alkyl substituents, leading to weaker hydration and lower overall hydrophilicity of the salts [54]. The effect is particularly pronounced with ethyl substitution, because the larger ethyl groups more effectively disrupt the surrounding water structure compared to methyl groups. This occurs even though EACl retains three N–H bonds capable of hydrogen bonding with water [62]. The Soret coefficient and its temperature sensitivity do not depend solely on hydration. Many other factors, such as mass and moment of inertia, also play a role. For the salt investigated here, $\log D_{\textrm{pH}}$ and $M_w$ vary in a similar way, suggesting that the temperature sensitivity of the Soret coefficient may be related to mass. However, when compared with a larger selection of salts, the mass is no longer correlated with the logarithm of the partition coefficient [37].

**Fig. 7:**
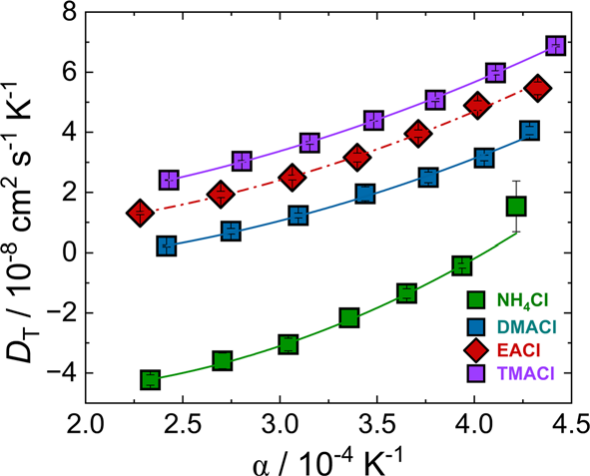
$D_{\textrm{T}}$ plotted as a function of the thermal expansion coefficient $\alpha$ for all ammonium salts at a concentration of 1 mol/kg. The lines are quadratic fits to the data

### Correlation between $D_{\textrm{T}}$ and the thermal expansion coefficient $\alpha$

A noteworthy finding from experiments is the linear correlation between the thermal diffusion coefficient $D_{\mathrm T}$ and the thermal expansion coefficient $\alpha$ for both polar and nonpolar mixtures. Different theoretical models in the literature relate the thermophoretic mobility and the thermal diffusion coefficient to the thermal expansion coefficients $\alpha$ or the ratio of $\alpha$ over viscosity [63, 64]. This can be understood intuitively: a higher coefficient of thermal expansion leads to greater thermal mobility, whereas higher viscosity slows movement down, resulting in lower mobility. Experimentally, a linear correlation between $D_{\textrm{T}}$ and $\alpha$ has been observed in nonpolar organic mixtures and aqueous solutions of non-ionic solutes [20, 65–67].

In Fig. 7, we show the thermal diffusion coefficients of aqueous ammonium salt solutions (1 mol/kg) versus the thermal expansion coefficient $\alpha =(1/V)\cdot (\partial V/\partial T)$. The effective volume, *V*, has been estimated using the Clausius–Mossotti equation from the refractive index measurements, leading to the thermal expansion coefficient [20]

5 $$\begin{aligned} \alpha \left( T \right) = - \frac{{6 \cdot n\left( T \right) \cdot \left( {{{\partial n}}/{{\partial T}}} \right) _{p,c}}}{{n{{\left( T \right) }^4} + n{{\left( T \right) }^2} - 2}} \end{aligned}$$

In contrast with aqueous solutions of non-ionic solutes, we find small deviations from a linear relationship between $D_{\textrm{T}}$ and $\alpha$. Further information on the correlation between the Soret coefficient $S_{\textrm{T}}$ and $\alpha$ and the data for 4 mol/kg can be found in Section S6 in the supplementary information. Apart from minor deviations from a linear relationship, we confirm the proportionality between $D_{\textrm{T}}$ and $\alpha$ for aqueous electrolyte solutions, as was observed for nonpolar organic mixtures and aqueous solutions of non-ionic solutes [20, 65–67].

## Conclusion

In this study we have investigated the thermophoretic properties of ammonium chloride and its alkyl- substituted derivatives (DMACl, EACl, and TMACl) in aqueous solution as function of temperature and concentration by IR-TDFRS to explore the influence of alkyl substitution on the Soret coefficient.

The concentration and temperature dependence of $S_{\textrm{T}}$ are strongly affected by the degree of substitution. While the hydrophilicity inferred from $\log D_{\textrm{pH}}$ values decreases with increasing alkylation, the sequence of $S_{\mathrm T}$ follows the same trend ($\hbox {NH}_4$Cl $\ll$ DMACl < EACl $\ll$ TMACl). The substituted ammonium salts display a systematic increase of $S_{\textrm{T}}$ with temperature, and for DMACl, EACl, and TMACl, a monotonic decrease with concentration. TMACl exhibits positive $S_{\textrm{T}}$ and thermophobic behavior across the entire concentration range studied, while DMACl and EACl remain thermophobic over a large range but become thermophilic at low concentrations and temperatures. In contrast, $\hbox {NH}_4$Cl exhibits predominantly thermophilic behavior under the studied conditions and shows a non-monotonic dependence on concentration at higher temperatures. A possible explanation is that, at lower temperatures, the minimum of the Soret coefficient occurs at concentrations below the experimentally accessible range. Such a concentration shift has been reported in the literature for LiCl and GdmCl [23, 39].

Furthermore, Eq. 4 can be used to describe the temperature dependence of $S_{\textrm{T}}$ for all concentrations. We observe a direct correlation between the temperature sensitivity of $S_{\mathrm T}$, $\Delta S_{\mathrm T}(\Delta T)$, and $\log D_{\textrm{pH}}$, revealing that the most hydrophilic salt ($\hbox {NH}_4$Cl) exhibits the strongest response to temperature changes. Meanwhile, alkyl substitution alters both the hydrophilicity and the thermodiffusive behavior. For these salts, $\Delta S_{\mathrm T}(\Delta T)$ values decrease with a decrease in hydrophilicity, following the trend: $\hbox {NH}_4$Cl > DMACl > EACl > TMACl.

Moreover, the thermal diffusion coefficient, $D_{\mathrm T}$, exhibits a proportional correlation with the thermal expansion coefficient, $\alpha$, for all ammonium salts investigated. In contrast with aqueous solutions of non-ionic solutes, deviations from a strictly linear relationship between $D_{\mathrm T}$ and $\alpha$ are observed, with a quadratic dependence providing a better description of the data. Nevertheless, the overall proportionality between $D_{\mathrm T}$ and $\alpha$ remains consistent with trends reported for nonpolar organic mixtures and aqueous solutions of non-ionic solutes.

Our results extend the understanding of ion-specific thermodiffusion by highlighting the role of alkyl substitution in ammonium salts. As ammonium derivatives are widely applied as intermediates in pharmaceutical synthesis and in soft-matter systems, the correlations established here may serve as a basis for tailoring thermophoretic transport in complex environments.

## Supplementary Information

Below is the link to the electronic supplementary material.

Supplementary file 1 (pdf 3473 KB)

## Data Availability

The data that support the findings of this study are available from Zenodo https://doi.org/10.5281/zenodo.18174547.

## References

[1] S. Xu, J.F. Torres, All-liquid thermal desalination and brine concentration via multichannel thermodiffusion. Nat. Water **3**, 617–631 (2025)

[2] M.H. Nikpoor, M. Dejam, Z. Chen, M. Clarke, Chemical-gravity-thermal diffusion equilibrium in two-phase non-isothermal petroleum reservoirs. Energy Fuels **30**, 2021–2034 (2016)

[3] A. Floroni, N. Yeh Martín, T. Matreux, L.I. Weise, S.S. Mansy, H. Mutschler, C.B. Mast, D. Braun, Membraneless protocell confined by a heat flow. Nat. Phys. **21**, 1303–1310 (2025) 40814307 10.1038/s41567-025-02935-4 PMC12343290

[4] B.T. Huang, M. Roger, M. Bonetti, T.J. Salez, C. Wiertel-Gasquet, E. Dubois, R. Cabreira Gomes, G. Demouchy, G. Mériguet, V. Peyre, M. Kouyaté, C.L. Filomeno, J. Depeyrot, F.A. Tourinho, R. Perzynski, S. Nakamae, Thermoelectricity and thermodiffusion in charged colloids. J. Chem. Phys. **143**, 054902 (2015) 26254665 10.1063/1.4927665

[5] I.C. Perez de Luco, A. Errarte, P.F. Arroiabe, A. Mialdun, A. Mojtabi, P. Costeseque, V. Shevtsova, M.M. Bou-Ali, Mass transport in libr-h2o solutions: coupling between diffusion, thermodiffusion, and composition. J. Chem. Phys. **163**, 034501 (2025) 40662719 10.1063/5.0276430

[6] P. Baaske, F.M. Weinert, S. Duhr, K.H. Lemke, M.J. Russell, D. Braun, Extreme accumulation of nucleotides in simulated hydrothermal pore systems. Proc. Natl. Acad. Sci. U.S.A. **104**, 9346–9351 (2007) 17494767 10.1073/pnas.0609592104 PMC1890497

[7] S.A.I. Seidel, P.M. Dijkman, W.A. Lea, G. van den Bogaart, M. Jerabek-Willemsen, A. Lazic, J.S. Joseph, P. Srinivasan, P. Baaske, A. Simeonov, I. Katritch, F.A. Melo, J.E. Ladbury, G. Schreiber, A. Watts, D. Braun, S. Duhr, Microscale thermophoresis quantifies biomolecular interactions under previously challenging conditions. Methods **59**, 301–315 (2013) 10.1016/j.ymeth.2012.12.005 PMC3644557 23270813

[8] D. Niether, S. Di Lecce, F. Bresme, S. Wiegand, Unravelling the hydrophobicity of urea in water using thermodiffusion: implications for protein denaturation. Phys. Chem. Chem. Phys. **20**, 1012–1020 (2018) 29235590 10.1039/c7cp05843h

[9] D. Niether, S. Wiegand, Thermophoresis of biological and biocompatible compounds in aqueous solution. J. Phys.: Condes. Matter **31**, 503003 (2019) 10.1088/1361-648X/ab421c 31491783

[10] S. Mohanakumar, H. Kriegs, W.J. Briels, S. Wiegand, Overlapping hydration shells in salt solutions causing non-monotonic Soret coefficients with varying concentration. Phys. Chem. Chem. Phys. **24**, 27380–27387 (2022) 36331005 10.1039/d2cp04089a

[11] B.A. Rudani, S. Docter, S. Schott-Verdugo, J. Buitenhuis, A.M. Stadler, H. Gohlke, S. Wiegand, Influence of -helical content on the thermodiffusion of apomyoglobin. Langmuir **41**, 28322–28334 (2025) 41091567 10.1021/acs.langmuir.5c02797 PMC12573798

[12] S. Iacopini, R. Rusconi, R. Piazza, The macromolecular tourist: universal temperature dependence of thermal diffusion in aqueous colloidal suspensions. Eur. Phys. J. E **19**, 59–67 (2006) 16446985 10.1140/epje/e2006-00012-9

[13] H. Ning, J.K.G. Dhont, S. Wiegand, Thermal-diffusive behavior of a dilute solution of charged colloids. Langmuir **24**, 2426–2432 (2008) 18254649 10.1021/la703517u

[14] Z. Wang, H. Kriegs, J. Buitenhuis, J.K.G. Dhont, S. Wiegand, Thermophoresis of charged colloidal rods. Soft Matter **9**, 8697–8704 (2013)

[15] R. Kita, S. Wiegand, Soret coefficient of poly(n-isopropylacrylamide)/water in the vicinity of coil-globule transition temperature. Macromolecules **38**, 4554–4556 (2005)

[16] Y. Kishikawa, S. Wiegand, R. Kita, Temperature dependence of Soret coefficient in aqueous and nonaqueous solutions of pullulan. Biomacromol **11**, 740–747 (2010) 10.1021/bm9013149 20121135

[17] Z. Wang, D. Afanasenkau, M. Dong, D. Huang, S. Wiegand, Molar mass and temperature dependence of the thermodiffusion of polyethylene oxide in water/ethanol mixtures. J. Chem. Phys. **141**, 064904–10649047 (2014) 25134596 10.1063/1.4891720

[18] Z. Wang, H. Kriegs, S. Wiegand, Thermal diffusion of nucleotides. J. Phys. Chem. B **116**, 7463–7469 (2012) 22663072 10.1021/jp3032644

[19] D. Niether, M. Sarter, B.W. Koenig, J. Fitter, A.M. Stadler, S. Wiegand, Thermophoresis: the case of streptavidin and biotin. Polymers **12**, 376 (2020) 32046223 10.3390/polym12020376 PMC7077373

[20] D. Niether, H. Kriegs, J.K.G. Dhont, S. Wiegand, Peptide model systems: correlation between thermophilicity and hydrophilicity. J. Chem. Phys. **149**, 044506 (2018) 30068171 10.1063/1.5042051

[21] A.L. Sehnem, D. Niether, S. Wiegand, A.M. Figueiredo Neto, Thermodiffusion of monovalent organic salts in water. J. Phys. Chem. B **122**, 4093–4100 (2018) 29558136 10.1021/acs.jpcb.8b01152

[22] S. Mohanakumar, J. Luettmer-Strathmann, S. Wiegand, Thermodiffusion of aqueous solutions of various potassium salts. J. Chem. Phys. **154**, 084506 (2021) 33639776 10.1063/5.0038039

[23] B.A. Rudani, A. Jakubowski, H. Kriegs, S. Wiegand, Deciphering the guanidinium cation: insights into thermal diffusion. J. Chem. Phys. **160**, 214502 (2024) 38828819 10.1063/5.0215843

[24] I. Skarmoutsos, E. Guardia, Solvation structure and dynamics of the dimethylammonium cation diluted in liquid water: a molecular dynamics approach. J. Chem. Phys. **152**, 234501 (2020) 32571039 10.1063/5.0004204

[25] Z. Jing, Y. Zhou, T. Yamaguchi, K. Yoshida, K. Ikeda, K. Ohara, G. Wang, Hydration of alkali metal and halide ions from static and dynamic viewpoints. J. Phys. Chem. Lett. **14**, 6270–6277 (2023) 37399074 10.1021/acs.jpclett.3c01302

[26] K.P. Gregory, E.J. Wanless, G.B. Webber, V.S.J. Craig, A.J. Page, The electrostatic origins of specific ion effects: quantifying the Hofmeister series for anions. Chem. Sci. **12**, 15007–15015 (2021) 34976339 10.1039/d1sc03568a PMC8612401

[27] A. Lileev, D. Loginova, A. Lyashchenko, L. Timofeeva, N. Kleshcheva, The hydrophobic hydration in aqueous solutions of allyl-substituted ammonium salts. J. Mol. Liq. **131–132**, 101–104 (2007)

[28] J. Teychené, H. Roux-de Balmann, L. Maron, S. Galier, Investigation of ions hydration using molecular modeling. J. Mol. Liq. **294**, 111394 (2019)

[29] D. Laage, T. Elsaesser, J.T. Hynes, Water dynamics in the hydration shells of biomolecules. Chem. Rev. **117**, 10694–10725 (2017) 28248491 10.1021/acs.chemrev.6b00765 PMC5571470

[30] C.C. Tanner, The Soret effect. Part 1. Trans. Faraday Soc. **23**, 75–95 (1927)

[31] P.N. Snowdon, J.C.R. Turner, The concentration dependence of the Soret effect. Trans. Faraday Soc. **56**, 1812–1819 (1960)

[32] F. Römer, Z. Wang, S. Wiegand, F. Bresme, Alkali halide solutions under thermal gradients: Soret coefficients and heat transfer mechanisms. J. Phys. Chem. B **117**, 8209–8222 (2013) 23758489 10.1021/jp403862x

[33] M. Jokinen, J.A. Manzanares, K. Kontturi, L. Murtomäki, Thermal potential of ion-exchange membranes and its application to thermoelectric power generation. J. Membr. Sci. **499**, 234–244 (2016)

[34] S. Di Lecce, T. Albrecht, F. Bresme, The role of ion-water interactions in determining the Soret coefficient of LiCl aqueous solutions. Phys. Chem. Chem. Phys. **19**, 9575–9583 (2017) 28345697 10.1039/c7cp01241a

[35] L. Rezende Franco, A.L. Sehnem, A.M. Figueiredo Neto, K. Coutinho, Molecular dynamics approach to calculate the thermodiffusion (Soret and Seebeck) coefficients of salts in aqueous solutions. JCTC **17**, 3539–3553 (2021) 33942620 10.1021/acs.jctc.1c00116

[36] A.J. Hutchinson, J.F. Torres, B. Corry, Modeling thermodiffusion in aqueous sodium chloride solutions–Which water model is best? J. Chem. Phys. **156**(16), 164503 (2022) 35490021 10.1063/5.0088325

[37] S. Mohanakumar, S. Wiegand, Towards understanding specific ion effects in aqueous media using thermodiffusion. Eur. Phys. J. E **45**, 10 (2022) 35106668 10.1140/epje/s10189-022-00164-8 PMC8807466

[38] F. Bresme, E. Vasey, Thermal transport of alkali halide aqueous solutions: a non-equilibrium molecular dynamics investigation. Mol. Phys. **122**, 2388302 (2024) 10.3390/e27020193 PMC11853856 40003190

[39] N. Lee, S. Mohanakumar, W.J. Briels, S. Wiegand, Non-monotonic Soret coefficients of aqueous LiCl solutions with varying concentrations. Phys. Chem. Chem. Phys. **26**, 7830–7836 (2024) 38375894 10.1039/d3cp06061f

[40] B.A. Rudani, W.J. Briels, S. Wiegand, Analyzing the concentration-dependent Soret coefficient minimum in salt solutions: an overview. Phys. Chem. Chem. Phys. **27**, 4746–4755 (2025) 39946123 10.1039/d4cp04477k

[41] J. Colombani, J. Bert, J. Dupuy-Philon, Thermal diffusion in (LiCl, RH2O). J. Chem. Phys. **110**, 8622–8627 (1999)

[42] V.V. Goncharuk, E.A. Orekhova, V.V. Malyarenko, Influence of temperature on water clusters. J. Water Chem. Technol. **30**, 80–84 (2008)

[43] F. D’Amico, F. Bencivenga, A. Gessini, C. Masciovecchio, Temperature dependence of hydrogen-bond dynamics in acetic acidwater solutions. J. Phys. Chem. B **114**, 10628–10633 (2010) 20701390 10.1021/jp103730s

[44] I. Aliagas, A. Gobbi, M.-L. Lee, B.D. Sellers, Comparison of logp and logd correction models trained with public and proprietary data sets. J. Comput. Aided Mol. Des. **36**, 253–262 (2022) 35359246 10.1007/s10822-022-00450-9

[45] F.S. Gaeta, G. Perna, G. Scala, F. Bellucci, Non-isothermal matter transport in sodium-chloride and potassium chloride aqueous solutions. 1. Homogeneous system (thermal-diffusion). J. Phys. Chem. **86**, 2967–2974 (1982)

[46] J. Colombani, H. Dez, J. Bert, J. Dupuy-Philon, Hydrodynamic instabilities and Soret effect in an aqueous electrolyte. Phys. Rev. E **58**, 3202–3208 (1998)

[47] O.R. Gittus, F. Bresme, Mass dipole contribution to the isotopic Soret effect in molecular mixtures. J. Chem. Phys. **159**, 114503 (2023) 37724736 10.1063/5.0164253

[48] J. Chanu, Etude de leffet Soret dans les solutions ioniques. 4. les resultats. J. Chim. Phys.-Chim. Biol. **55**, 743–753 (1958)

[49] J.N. Agar, J.C.R. Turner, Thermal diffusion in solutions of electrolytes. Proc. R. Soc. Lond. Ser. A, Math. Phys. Sci. **255**, 307–330 (1960)

[50] C. D. Price, Thermal diffusion in solutions of electrolytes, Tech. Rep. U.S.A.F, Contract No. AF(052)-99 Accession number AD0276280. Report date 1961 Dec 18

[51] O.R. Gittus, F. Bresme, On the microscopic origin of Soret coefficient minima in liquid mixtures. Phys. Chem. Chem. Phys. **25**, 1606–1611 (2023) 36541658 10.1039/d2cp04256h

[52] M. Ekimova, W. Quevedo, Ł Szyc, M. Iannuzzi, P. Wernet, M. Odelius, E.T.J. Nibbering, Aqueous solvation of ammonia and ammonium: probing hydrogen bond motifs with ft-ir and soft x-ray spectroscopy. J. Am. Chem. Soc. **139**, 12773–12783 (2017) 28810120 10.1021/jacs.7b07207

[53] D. Das Mahanta, N. Samanta, R.K. Mitra, Decisive role of hydrophobicity on the effect of alkylammonium chlorides on protein stability: A terahertz spectroscopic finding. J. Phys. Chem. B **121**, 7777–7785 (2017) 28742966 10.1021/acs.jpcb.7b04088

[54] J.B. Macaskill, R.G. Bates, Osmotic and activity coefficients of monomethyl-, dimethyl-, and trimethylammonium chlorides at c. J. Solution Chem. **15**, 323–330 (1986)

[55] S. Wiegand, H. Ning, H. Kriegs, Thermal diffusion forced rayleigh scattering setup optimized for aqueous mixtures. J. Phys. Chem. B **111**, 14169–14174 (2007) 18052276 10.1021/jp076913y

[56] K. Thyagarajan, P. Lallemand, Determination of thermal-diffusion ratio in a binary mixture by forced rayleigh-scattering. Opt. Commun. **26**, 54–57 (1978)

[57] W. Köhler, P. Rossmanith, Aspects of thermal-diffusion forced rayleigh-scattering - heterodyne-detection, active phase tracking, and experimental constraints. J. Phys. Chem. **99**, 5838–5847 (1995)

[58] J.K. Platten, M.M. Bou-Ali, J.F. Dutrieux, Precise determination of the Soret, thermodiffusion and isothermal diffusion coefficients of binary mixtures of dodecane, isobutylbenzene and 1,2,3,4-tetrahydronaphthalene (contribution of the university of mons to the benchmark test). Philos. Mag. **83**, 2001–2010 (2003)

[59] H. Hoang, G. Galliero, Predicting thermodiffusion in simple binary fluid mixtures. Eur. Phys. J. E **45**, 42 (2022) 35507140 10.1140/epje/s10189-022-00197-z

[60] M. Stefan-Kharicha, A. Kharicha, J. Mogeritsch, M. Wu, A. Ludwig, Review of ammonium chloride-water solution properties. J. Chem. Eng. Data **63**, 3170–3183 (2018)

[61] G. Zhao, F. Bresme, Thermal transport anomalies of electrolyte solutions in the water supercooled regime: signatures of the liquid-liquid water phase transition. J. Chem. Phys. **163**, 224505 (2025) 41363752 10.1063/5.0299902

[62] V. Migliorati, P. Ballirano, L. Gontrani, A. Triolo, R. Caminiti, Thermal and structural properties of ethylammonium chloride and its mixture with water. J. Phys. Chem. B **115**, 4887–4899 (2011) 21486048 10.1021/jp2010138

[63] S. Semenov, M. Schimpf, Thermophoresis of dissolved molecules and polymers: consideration of the temperature-induced macroscopic pressure gradient. Phys. Rev. E **69**, 011201 (2004) 10.1103/PhysRevE.69.011201 14995607

[64] H. Brenner, Elementary kinematical model of thermal diffusion in liquids and gases. Phys. Rev. E **74**, 036306 (2006) 10.1103/PhysRevE.74.036306 17025742

[65] P. Blanco, M.M. Bou-Ali, J.K. Platten, P. Urteaga, J.A. Madariaga, C. Santamaria, Determination of thermal diffusion coefficient in equimolar n-alkane mixtures: empirical correlations. J. Chem. Phys. **129**, 174504 (2008) 19045355 10.1063/1.2945901

[66] P. Blanco, S. Wiegand, Study of the soret effect in monosaccharide solutions. J. Phys. Chem. B **114**, 2807–2813 (2010) 10.1021/jp910331a 20136092

[67] P. Blanco, H. Kriegs, B. Arlt, S. Wiegand, Thermal diffusion of oligosaccharide solutions: the role of chain length and structure. J. Phys. Chem. B **114**, 10740–10747 (2010) 20684655 10.1021/jp104534m

