# Supplementary material for: Hydrophilicity controls thermodiffusion in alkylammonium chlorides
Source: Eur Phys J E Soft Matter. 2026 Feb 24;49(3):17. doi: 10.1140/epje/s10189-026-00561-3 (PMC12932385; doi:10.1140/epje/s10189-026-00561-3)
Supplement: Supplementary file 1 — (pdf 3473 KB) [file 10189_2026_561_MOESM1_ESM.pdf]

Supplementary Information: Hydrophilicity Controls Thermodiffusion in Alkylammonium Chlorides

Binny A. Rudani, Hartmut Kriegs
and Simone Wiegand

Contents

S1 Refractive index contrast measurements **S2**

S2 Thermal diffusion and diffusion coefficient for aqueous ammonium salt solutions **S5**

S3 Calculated $\log D_{\mathrm{pH}}$-values **S8**

S4 Temperature dependence of S_{T} for DMACl **S8**

S5 Temperature sensitivity of S_{T} **S9**

S6 Relation between thermal diffusion properties and thermal expansion coefficient **S10**

S1 Refractive index contrast measurements

The refractive index contrast factors with concentration required to calculate S_T were measured using an Abbe refractometer (Anton Paar Abbemat MW) at a wavelength of 632.8 nm. For each salt, the refractive index was measured at five concentrations around the desired concentration at seven temperatures ranging from 15 to 45°C. The concentration dependence, $(\partial n/\partial c)_{p,T}$, was determined from the slope of a linear fit to these data. Figure S1 shows the slight decrease in the refractive index increment $(\partial n/\partial c)_{p,T}$ with temperature and the increase of $(\partial n/\partial c)_{p,T}$ with concentration for the investigated aqueous ammonium salt solutions.

The refractive index increment with temperature $(\partial n/\partial T)_{p,c}$ was measured interferometrically [1]. Measurements were performed over a temperature range from 15 to 45°C. The solution was heated and cooled automatically at a rate of 1.1 mK/s. In all cases, the refractive index increment varies linearly with both concentration and temperature within the investigated range. Figure S2 shows the decrease of the refractive index increment $(\partial n/\partial T)_{p,c}$ with temperature and the decrease of $(\partial n/\partial T)_{p,c}$ with concentration for aqueous ammonium salt solutions. For NH_4Cl, however, the concentration trend reverses at 45°C, and the $(\partial n/\partial T)_{p,c}$ value increases as the concentration increases. While the increment is nearly independent of temperature at 40°C.

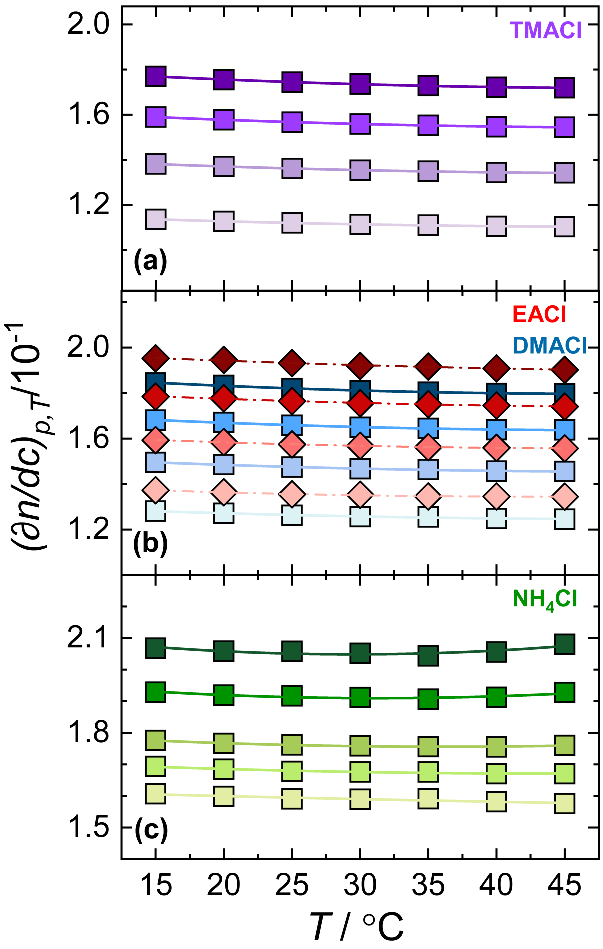

Figure S1: Temperature dependence of $(\partial n/\partial c)_{p,T}$, for aqueous ammonium salt solutions at concentrations ranging from 1 to 4 mol/kg. Symbol darkness increases with concentration, from light (1 mol/kg) to dark (4 mol/kg).

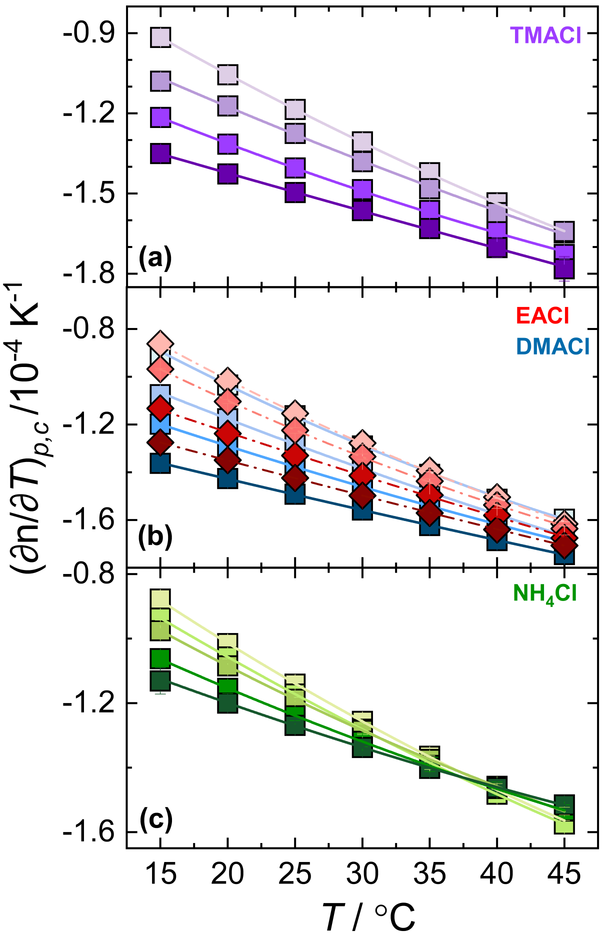

Figure S2: Temperature dependence of $(\partial n/\partial T)_{p,c}$, for aqueous ammonium salt solutions at concentrations ranging from 1 to 4 mol/kg. Symbol darkness increases with concentration, from light (1 mol/kg) to dark (4 mol/kg).

S2 Thermal diffusion and diffusion coefficient for aqueous ammonium salt solutions

The temperature-dependent behavior of D_T for aqueous ammonium salt solutions at different concentrations is shown in Fig. S3. All salts investigated here showed similar behaviour as S_T. At a given concentration, D_T increased with temperature. However, at a fixed temperature, D_T generally decreased with increasing salt concentration. An exception was observed for NH_4Cl, which showed a non-monotonic trend, exhibiting a minimum in D_T with added salt concentration, as shown in the inset.

The temperature-dependent behavior of the D for aqueous ammonium salt solutions at varying concentrations is presented in Fig. S4. Across all salts studied, D consistently increases with temperature, a trend primarily attributed to the reduction in solution viscosity at higher temperatures.

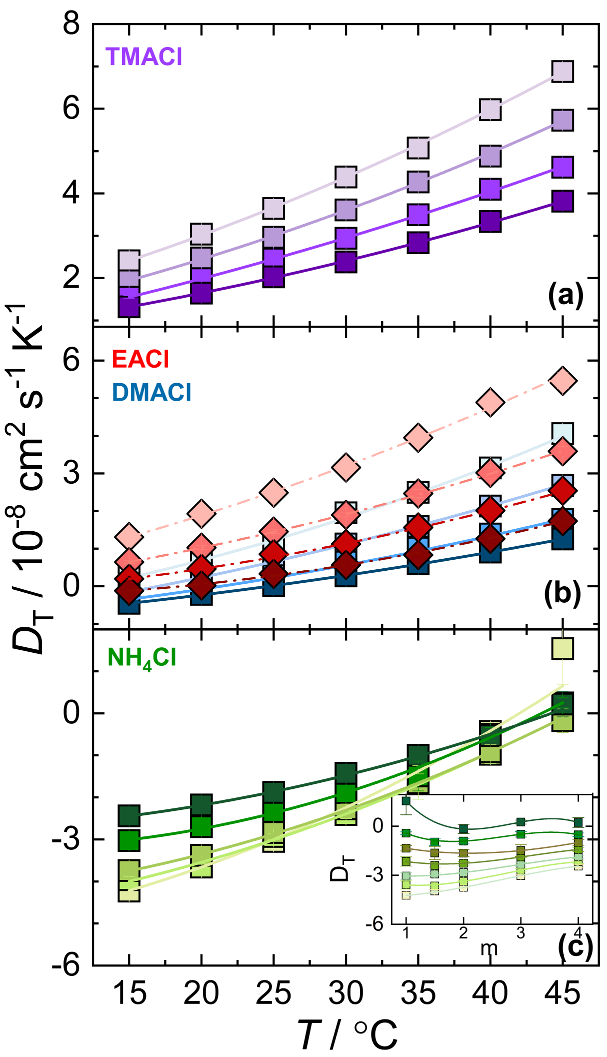

Figure S3: Thermal diffusion coefficient of ammonium salts as a function of temperature. Symbol darkness increases with concentration, from light (1 mol/kg) to dark (4 mol/kg). Lines are included to guide the eye.

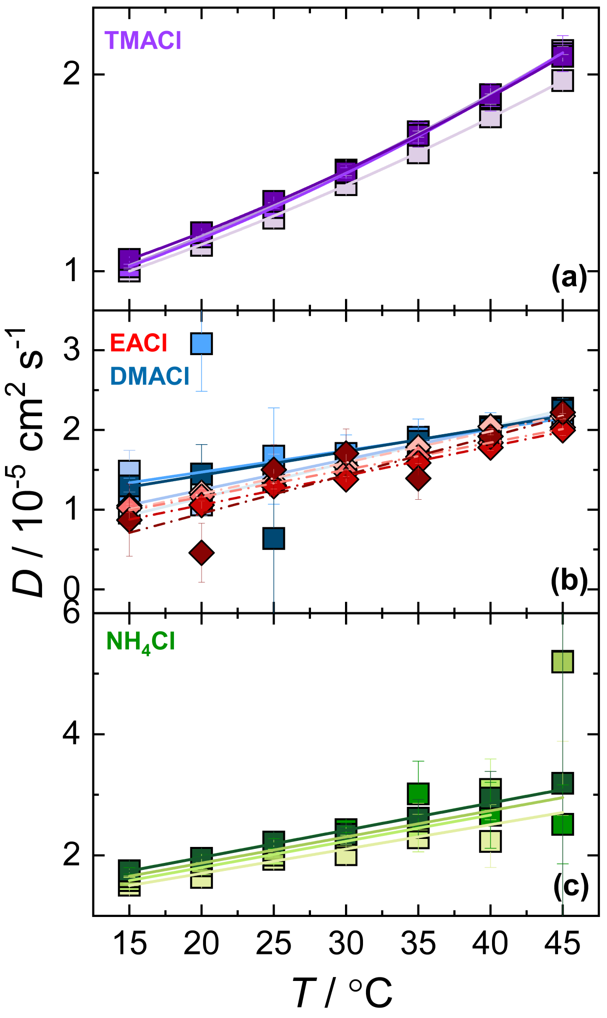

Figure S4: Diffusion coefficient of ammonium salts as a function of temperature. Symbol darkness increases with concentration, from light (1 mol/kg) to dark (4 mol/kg). Lines are included to guide the eye.

S3 Calculated $\log D_{\mathrm{pH}}$-values

The $\log D_{\mathrm{pH}}$ values were computed using the Marvin 24.1.2 software from ChemAxon (http://www.chemaxon.com), employing calculator plugins based on the methodology described by Viswanadhan et al. [2]. For these calculations, specific parameters were defined to ensure consistency: the concentrations of Na^+ and K^+ ions were set to 0 mol/dm^3, while the Cl^- concentration was fixed at 1 mol/dm^3. The calculation was performed at the pH measured at 1 M salt concentration, selecting the corresponding $\log D_{\mathrm{pH}}$ value for that pH. To validate this approach, we also measured the pH of salt solutions at various concentrations (0.02 M, 0.1 M, and 2 M), which exhibited the same trend in $\log D_{\mathrm{pH}}$ as observed at 1 M. Table 1 in the main manuscript tabulates the resulting $\log D_{\mathrm{pH}}$ values for all salts studied. The interpretation of $\log D_{\mathrm{pH}}$ is similar to that of $\log P$. More negative values indicate an increased hydrophilicity and reflect a stronger affinity of the compound for the aqueous phase [3].

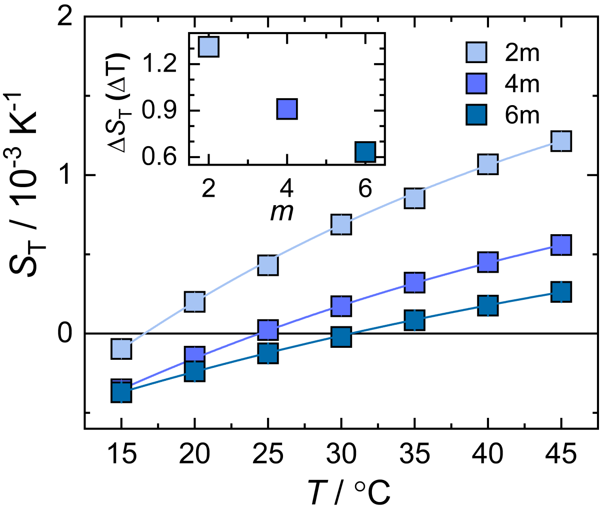

Figure S5: Soret coefficient of DMACl as a function of temperature. Symbol darkness increases with concentration, from light (2 mol/kg) to dark (6 mol/kg). Lines are fit according to Eq. 4 in the main manuscript.

S4 Temperature dependence of S_T for DMACl

We extended the study further to a higher concentration of 6 mol/kg of DMACl to investigate whether S_T increases at higher concentrations. Figure

S5 shows the variation of S_T with temperature for different concentrations, while the inset shows the temperature sensitivity, defined as $\Delta S_T(\Delta T) = S_T(45°C) - S_T(15°C)$, plotted against concentration. The results reveal that S_T increases consistently with both concentration and temperature, without any noticeable deviation from the overall trend. Contrarily, $\Delta S_T(\Delta T)$ decreases progressively with increasing concentration.

Table S1: Summary of fitting parameters derived from Eq. 4 for ammonium salt solutions across all investigated concentrations.

Salt	m / mol/kg	$S_T^\infty/10^{-3}$ K^{-1}	$-A/10^{-3}$ K^{-1}	T^0 / °C
NH_4Cl	1	3.25 ± 0.57	8.52 ± 0.42	44.11 ± 5.67
NH_4Cl	1.5	3.05 ± 0.80	7.53 ± 0.64	49.54 ± 9.05
NH_4Cl	2	2.39 ± 0.07	6.44 ± 0.05	46.32 ± 0.82
NH_4Cl	3	13.07 ± 5.77	15.77 ± 5.7	230.55 ± 97.8
NH_4Cl	4	2.39 ± 0.29	4.8 ± 0.26	63.87 ± 6.08
DMACl	1	2.86 ± 0.15	4.09 ± 0.08	33.9 ± 2.73
DMACl	2	2.30 ± 0.06	3.56 ± 0.04	37.76 ± 1.22
DMACl	3	1.95 ± 0.19	3.11 ± 0.13	44.16 ± 5.55
DMACl	4	1.56 ± 0.08	2.64 ± 0.06	46.31 ± 2.39
DMACl	6	1.17 ± 0.11	2.0 ± 0.1	57.42 ± 5.18
EACl	1	3.11 ± 0.10	3.25 ± 0.04	26.79 ± 2.43
EACl	2	2.79 ± 0.08	3.15 ± 0.05	39.9 ± 2.2
EACl	3	2.59 ± 0.26	3.16 ± 0.2	51.49 ± 7.5
EACl	4	1.73 ± 0.60	2.73 ± 0.42	41.95 ± 18.62
TMACl	1	4.34 ± 0.32	2.87 ± 0.18	35.96 ± 9
TMACl	2	3.18 ± 0.11	2.11 ± 0.06	30.64 ± 4.09
TMACl	3	2.54 ± 0.10	1.82 ± 0.04	26.09 ± 3.87
TMACl	4	2.11 ± 0.07	1.5 ± 0.02	27.95 ± 3.7

S5 Temperature sensitivity of S_T

We successfully described the temperature dependence of S_T for the investigated salt solutions using Eq. 4 in the main manuscript. The obtained parameters are shown in Table S1. For a few fits, the uncertainty of the fitted parameter reaches 44%. If we exclude these, the typical uncertainty of S_T^∞ and T_0 is about 15% and that of the amplitude A is about 10%. Large uncertainties of the fitted parameter occur especially, when the temperature dependence of S_T is nearly linear. Therefore we introduced in the main manuscript $\Delta S_T(\Delta T) = S_T$ (45°C)- S_T(15°C) as a measure of temperature sensitivity. $\Delta S_T(\Delta T)$ decreases with increasing concentration and with de-

creasing hydrophilicity of the solute molecules. The magnitude of A (see Table S1 and Figure S6) exhibits a similar trend, although the data are noisier than for $\Delta S_\mathrm{T}(\Delta T)$ and we do have an outlier for NH_4Cl at $m = 3$ mol/kg.

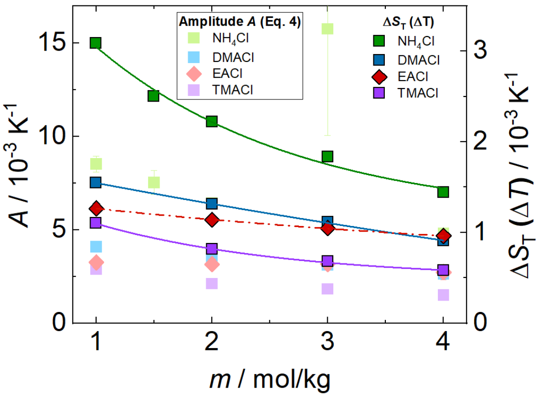

Figure S6: Amplitude A (plotted on the left axis and light color) and the change in the temperature sensitivity $\Delta S_\mathrm{T}(\Delta T)$ (plotted on the right axis and in dark color) are plotted as a function of increasing ammonium salt concentration. Both quantities exhibit similar concentration dependence. Nevertheless, if the temperature dependence of S_T is almost linear then the uncertainty of A gets large up to 36%. In those case $\Delta S_\mathrm{T}(\Delta T)$ is a more robust parameter. The lines are guides for the eye.

S6 Relation between thermal diffusion properties and thermal expansion coefficient

Figure S7(a) and Fig. S7(b) show the thermal expansion coefficients D_T and the Soret coefficient S_T of all ammonium salts as a function of the thermal expansion coefficient α at the lowest and highest concentration of 1 (corresponding to 5-9 wt%) and 4 mol/kg (corresponding to 18-28 wt%). Both coefficients increase with α, but only S_T of the solutions with 1 mol/kg can be described by a linear fit as a function of α. Although the deviations from a linear fit are not huge, a quadratic fit is required in all other cases.

Figure S7(c) shows that at the low concentration all systems show almost the same thermal expansion coefficient, which indicates that α is still close to the value of water at this low solute concentration. At higher concentrations the temperature dependence of α decreases for all systems and the differences between the ammonium salts get larger. Similar trends were found for the

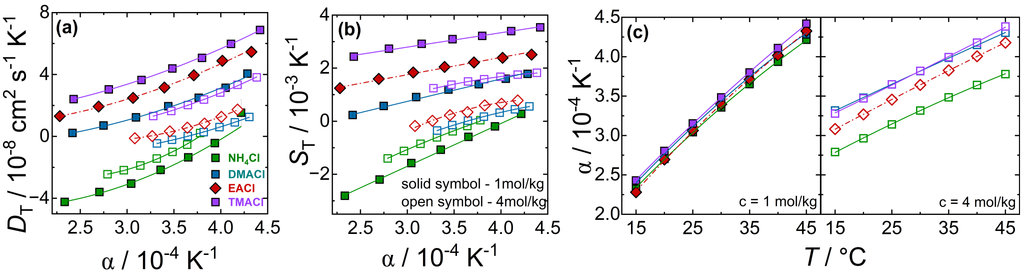

Figure S7: (a) The thermal diffusion coefficient and (b) the Soret coefficient of all ammonium salts as function of the thermal expansion coefficient α at the lowest (solid symbols) and highest (open symbols) concentration of 1 and 4 mol/kg, respectively. (c) Thermal expansion coefficient as a function of temperature at the lowest (left panel) and highest (right panel) concentration.

aqueous amide solutions [4].

References

[1] G. Wittko and W. Köhler. Precise determination of the Soret, thermal diffusion and mass diffusion coefficients of binary mixtures of dodecane, isobutylbenzene and 1,2,3,4-tetrahydronaphthalene by a holographic grating technique. *Philos. Mag.*, 83:1973–1987, 2003.

[2] V. N. Viswanadhan, A. K. Ghose, Ganapathi R. Revankar, and R. K. Robins. Atomic physicochemical parameters for three dimensional structure directed quantitative structure-activity relationships. 4. additional parameters for hydrophobic and dispersive interactions and their application for an automated superposition of certain naturally occurring nucleoside antibiotics. *J. Chem. Inf. Model.*, 29:163–172, 1989.

[3] D. Niether and S. Wiegand. Thermophoresis of biological and biocompatible compounds in aqueous solution. *J. Phys.: Condes. Matter*, 31:503003, 2019.

[4] D. Niether, H. Kriegs, J. K. G. Dhont, and S. Wiegand. Peptide model systems: Correlation between thermophilicity and hydrophilicity. *J. Chem. Phys.*, 149:044506, 2018.
